# Musculoskeletal Pain: Current and Future Directions of Physical Therapy Practice

**DOI:** 10.1016/j.arrct.2023.100258

**Published:** 2023-02-01

**Authors:** Brona M. Fullen, Harriet Wittink, An De Groef, Morten Hoegh, Joseph G. McVeigh, Denis Martin, Keith Smart

**Affiliations:** a UCD School of Public Health, Physiotherapy and Sports Science, University College Dublin, Ireland; b Research Centre Healthy and Sustainable Living Utrecht University of Applied Sciences, The Netherlands; c Department of Rehabilitation Sciences, Leuven, Belgium; d Department of Rehabilitation Sciences and Physiotherapy MOVANT, University of Antwerp, Antwerp, Belgium; e Pain in Motion International Research Group, www.paininmotion.be; f Aalborg University, Faculty of Medicine, Department of Health Science and Technology, Denmark; g Discipline of Physiotherapy, School of Clinical Therapies, College of Medicine and Health, University College Cork, Ireland; h Centre for Rehabilitation, School of Health and Life Sciences, Teesside University, United Kingdom; i NIHR Applied Research Collaborative North East and North Cumbria, Cumbria, Northumberland, United Kingdom

**Keywords:** Biopsychosocial model, Musculoskeletal pain, Physical therapy modalities, Rehabilitation, Therapeutics

## Abstract

Musculoskeletal (MSK) pain is 1 of the most common problems managed by clinicians in MSK care. This article reviews current frameworks for the assessment and management of MSK pain within evidence-based physical therapy practice. Key considerations related to the biopsychosocial model of pain, evidence-based practice, assessment, treatment, physical activity/movement behavior, risk stratification, communication as well as patient education and self-management skills within physical therapy and physical and rehabilitation medicine are addressed. The future direction of MSK pain management is also discussed, including strategies to promote evidence-based practice, behavior change, social prescribing, and the use of technologies.

## Introduction

Physical therapy is clinically and cost effective in the assessment and management of musculoskeletal (MSK) disorders.^1^ Current MSK practice typically involves 3 components: education, exercise, and physical therapy. Evidence for each of the 3 component is limited, including the best way to provide them, and the emphasis that should be placed on each. An evidence-based biopsychosocial (BPS) approach with the active engagement of the patient in their own care is advocated.

This article reviews current frameworks such as evidence-based practice (EBP), the BPS model, risk stratification, and psychologically-informed physical therapy for the assessment and management of MSK pain.

## Evidence-based practice

EBP has been defined as “the conscientious, explicit, and judicious use of current best evidence in making decisions about the care of individual patients” which includes the integration of best research evidence, clinical expertise, and patient values.^2^ It was subsequently pointed out that this definition did not include any reference to ethical principles. A definition of EBP for physiotherapists was suggested as “an area of study, research, and practice in which clinical decisions are based on the best available evidence, integrating professional practice and expertise with ethical principles.”^3^

Clinicians are in favor of EBP and research; however, it is not always used in clinical practice.^4^ Barriers to clinical use of EBP include lack of time and workload pressures, access to research, and difficulty translating research in to practice.^4^ Therefore, understanding, promoting, and maximizing the facilitators of EBP (postgraduate education, skills to engage in self-directed learning, beliefs that research and clinical guidelines can usefully inform clinical decision-making, and a willingness to change and adopt more effective methods) could be used to enhance clinical practice.^5^ Engaging in such strategies (educational interventions,^6^ training and education of stakeholders, adapting and tailoring the context, and supporting clinicians) have been shown to improve guideline adherence and knowledge. However, when evaluating resultant changes in patient-reported outcomes, the literature is very limited. A recent systematic review on allied health care professionals EBP training included 6 studies, of which only 3 including patient reported outcomes, with no significant changes reported. The authors conclude that either that the outcome measures were not sensitive, that different intervention strategies are needed to change patient outcomes, and that more research is needed in this area.^7^

## The biopsychosocial model

EBP within MSK care has been informed and shaped, in part, by the BPS model of illness and pain. The BPS model was presented in 1977 in response to perceived shortcomings of the biomedical model and as a means of acknowledging the biological, psychological, and social factors determinants of health and disease. It was proposed as “a blueprint for research, a framework for teaching, and a design for action in the real world of health care”.^8^ Crucially, it acknowledged the reality that illnesses and diseases are human experiences as much as pathologic entities.

### Application of the biopsychosocial model in clinical practice

MSK pain disorders are no longer considered a purely biomedical problem, but considered a complex problem which can be influenced by a wide range of other factors. These include cognitive, psychological, social, as well as biomedical factors. Assessing and treating patients according to the BPS model has been recommended in a number of pain-related clinical guidelines relevant to clinicians.^9^ The BPS model also forms the basis of the World Health Organization's International Classification of Functioning, Disability and Health,^10^ suggesting widespread endorsement of the model. However, there seems to be varying levels of confidence and proficiency among clinicians regarding psychosocially oriented clinical knowledge and practice.^11^

### Psychologically informed treatment in physical therapy

A range of psychologically informed physical therapy interventions have been developed and evaluated.^12^ This approach blends conventional physical therapy treatments with cognitive-behavioral therapies, that acknowledge the influence of a person's thoughts, feeling and behaviors together with wider socio-economic contextual factors.^13^ Examples of such approaches include graded activity/exposure, cognitive-behavioral therapy and acceptance, and commitment interventions where the patient is facilitated to use acceptance as a way to deal with negative thoughts and feelings and commits to positive values-based goals.^14^ Reviews show that the clinical effects of such interventions on pain-related outcomes are inconsistent.^12^^,^^14^

## Musculoskeletal pain assessment

Physical therapists and physical and rehabilitation medicine physicians are frequently consulted for their assessment and treatment of disorders of which pain is often the dominant feature, and of course pain may be considered a disease in its own right.^15^

### Palpation, structural integrity, serious pathology

Assessment of low back pain (LBP) and MSK disorders in general should exclude specific pathologies (eg, fracture, infection, malignancy),^16^ Assessment of “red flags” is essential in many pain conditions and is used to identify risk of serious pathology,^17^ although individual red flags cannot reliably predict pathology.^18^^,^^19^ As an alternative or complementary approach to the use of nominal patho-anatomic diagnoses,^20^ mechanism-based approaches to the management of pain have been advocated.^21^ According to the International Association for the Study of Pain (IASP), there are 3 broad categories of pain mechanisms (nociceptive, neuropathic, and nociplastic) that may occur alone or in combination.^21^ Nociception provides a means of neural feedback that allows the central nervous system to detect and avoid noxious and potentially damaging stimuli in both active and passive settings. Neuropathic pain is caused by a lesion or disease of the somatosensory system, including peripheral fibers (Aβ, Aδ, and C fibers) and central neurons. Nociceptive mechanisms are assumed to drive the pain experience during and immediately after acute injuries. Likewise, nociceptive and neuropathic mechanisms are dominant in pathologies such as cancer and neuropathic pain, and nociplastic may be the dominant mechanism in some nonspecific and chronic pain presentations, such as chronic non-specific LBP and chronic widespread pain/fibromyalgia.

### Physical activity/movement behavior

Guidelines for the assessment and management of MSK pain recommend the promotion of active lifestyles with regular physical activity (PA) as a first line treatment.^9^ Both short and long-term benefits on the pain experience have been reported^22^^,^^23^ as well as positive effects on cardiovascular health, mood, stress, sleep quality of life, and sexual function.^24^^,^^25^

Within the BPS assessment of a person in pain an assessment of their PA levels should be undertaken in order to develop the most appropriate intensity and targeted individualized exercise programme.^26^ Subjective measures such as PA questionnaires, for example, Baecke physical activity questionnaires,^27^ electronic diaries^28^ have been commonly used, although more recently objective measures, such as accelerometers, are increasingly used to objectively measure performance of activities in people with LBP.^29^^,^^30^ Studies show only a weak^31^ to moderate correlation^32^ between self-reported PA and objectively measured PA (accelerometry) in individuals with chronic pain. Most people with chronic pain underestimate their level of PA. There is also a discrepancy between the association between subjectively or objectively measured PA and important outcome measures such as pain intensity, anxiety, and disability.^30^^,^^31^

An international consensus on the term “movement behavior” has been reached, which includes sedentary behavior, PA, and exercise.^32^ Movement behavior describes the 24-hour pattern of movement and non-movement patterns (including sleep). The term behavior refers to the choices a person makes in whether to move and how to move (frequency intensity, etc). Consequently, there is a need for objective measurement of movement behavior in people in pain. Considering the factors that modulate PA, including the quantity, type, psychosocial, and lifestyle factors, will help in the diagnosis and in the development of individualized treatment planning. It will also help in the monitoring and assessment of the effect of physical therapy treatment over time.

### Risk stratification

Management of LBP should include stratifying patients into homogenous groups based on risk stratification and offering targeted treatment, which results in better outcomes, and is now specifically recommended in the United Kingdom's National Institute for Health and Care Excellence (NICE) guidelines.^33^ The most widely known tool for this approach is the STarT Back Screening tool that allows clinicians to identify those who are at a low, medium, or high risk of poorer clinical outcomes due to potentially modifiable physical and psychological prognostic indicators (low mood, anxiety, catastrophizing, and fear avoidance) for persistent disabling symptoms.^34^ Management of low-risk patients consists of advice (pamphlets, information video) and PA with an emphasis on promoting appropriate levels of activity, including return to work. For medium-risk patients, management should consist of referral for standardized physical therapy, to address symptoms and function. High-risk patients should be referred for psychologically informed physical therapy, again to address symptoms and functional impairment in addition to psychosocial issues that may present a barrier to recovery.^34^ The efficacy of this approach has been established in Europe but has not been replicated in the US, illustrating successful implementation may vary in different health service settings.^35^ Given that up to one-third of primary care patients with LBP have dominant psychosocial risk factors,^34^ identifying and implementing an early effective care to patients’ level of risk of poorer outcomes is important.^36^

### Communication skills

Clear communication between clinicians and their patients is essential to facilitate active patient involvement in the assessment and management process. Patient-centered communication, including motivational interviewing skills, have been shown to improve patient satisfaction, build confidence, and improved health-related knowledge in those with chronic MSK pain.^37^^,^^38^^,^^39^

A patient-centered communication style is fundamental to achieving active patient engagement.^40^^,^^41^ Communicating with empathy, developing congruence of the clinician's and patient's goals and taking a positive approach to build a therapeutic alliance and facilitate shared-decision making are all essential.^42^^,^^43^ Adapting the communication style to the individual patient, having the ability to communicate using plain language, being cognizant of their health literacy level, speaking directly to the patient, listening actively, and asking appropriate questions are important features of patient-centered communication.^41^ Health literacy is the degree to which individuals have the capacity to obtain, process, and understand basic health information needed to make appropriate health decisions. Research indicates that health literacy in general in Europe may be inadequate^44^ and hamper effective self-management in patients with chronic pain.^45^ Barriers to effective communication such as demographic characteristics (socio-economic background, age, education level) should also be considered. Patient-centered communication requires the time to implement; however, the investment will result in increased effectiveness of the applied pain management strategies.^46^^,^^47^

## Musculoskeletal pain management

Following a thorough BPS assessment, a number of evidenced-based treatments and approaches may be used to manage MSK problems.

### Manual therapy

Manual therapy has been a core intervention for physical therapists treating patients with MSK complaints and is recommended as an adjunct or second line treatment in clinical guidelines for non-specific LBP.^48^ However, the rationale underpinning manual therapy has changed from a strict biomechanical paradigm (changing or influencing somatic tissues) to a neurophysiological one.^49^ In this paradigm, manual therapy may provide its pain-relieving effects via the well-established descending modulatory pathways in the central nervous system.^49^ While the exact mechanisms by which manual therapies affect the nociceptive system are unknown, it is clear that moderately painful pressure can lead to short-lasting pain inhibition (sometimes referred to as pain-inhibits-pain) in both humans and rodents.^50^ Furthermore, manual therapy is likely to have an influence on pain via more cognitive and contextual factors.^49^^,^^51^ These include the clinician's professionalism, mindset, and appearance; the patient's beliefs, experiences, and expectations about their diagnosis and treatment, the physiotherapist-patient relation during the appointment, the application of the manual therapy technique, even the overall impression of the clinic/hospital department.^51^ Viewing manual therapy through the lens of neuroscience not only provides a likely mechanism but may explain why different manual therapies appear to have similar effects on MSK problems such as LBP.^52^ Manual therapy may also provide the skilled clinician with a tool to engage non-verbally with the patient.^53^

### Electro-physical modalities

NICE^33^ reports that most of the evidence for the use of electro-physical modalities for chronic primary pain is of low to very low quality. The committee's view is that there was considerable uncertainty in the data, with little evidence of long-term outcomes, and much heterogeneity in practice. While laser therapy has the strongest preliminary evidence of benefit (quality of life and pain), it is not recommended until further research is undertaken.

The NICE committee also does not recommend transcutaneous electrical nerve stimulation (TENS) (lack of evidence of benefit), ultrasound (no evidence), and interferential therapy (no evidence). The most recent Cochrane review on TENS (an overview of systematic reviews including 9 reviews and 51 TENS-related randomized controlled trials, n=2895) equally is unable to conclude with any confidence that, in people with chronic pain, TENS is harmful, or beneficial, for pain control, disability, health-related quality of life, use of pain-relieving medicines, or global impression of change.^54^

With regard to transcranial magnetic stimulation, the findings from a Cochrane systematic review (38 trials, n=1225) indicates that repetitive transcranial magnetic stimulation of the motor cortex, but not the dorsolateral prefrontal cortex, may provide short‐term, but likely clinically unimportant improvements in chronic pain and quality of life (low to very low quality evidence).^55^

### Physical activity and exercise

Evidence supports the use of exercise and PA in the management of chronic diseases including painful conditions such as osteoarthritis, rheumatoid arthritis, and fibromyalgia.^56^ Incorporation of exercise and PA and their importance are usually introduced during initial individual appointments and continued through to structured exercise classes or as a component of a chronic pain rehabilitation program. Despite the physical, psychosocial, and social benefits achieving the PA guideline goal of 30 minutes of moderate PA, a day is challenging for patients living with pain. Although the frequency, intensity, type, and time (duration) parameters (F.I.T.T.) for specific conditions are known, there is increasing awareness that adherence long-term to exercise programs are affected by other factors including depression and other physical health problems.^57^ Evidence suggests that exercise behaviors are modifiable; therefore, motivational/behavioral change strategies should be incorporated into exercise interventions to enhance patients’ motivation and longer-term adherence.^58^^,^^59^ The choice of exercise therapy should be closely aligned with patients’ preferences and goals as enjoyment in and commitment to the type of exercise will help with long-term adherence.

Feedback on PA and exercise levels is a powerful behavioral change tool,^60^ and the use of technologies such as wearable biosensors integrated into clothing, shoes, watches, and smart phones that acquire, transmit, store, and retrieve health-related data could be used to monitor and augment individualized rehabilitation.^61^ A recent systematic review of controlled trials reported that these devices have promise in relation to increasing PA participation^62^ or to maintain PA levels after structured lifestyle interventions.^63^ Going forward this technology may also reduce traditional health care usage of face-to-face appointments for providing ongoing support.^64^^,^^65^

### Self-management skills

Facilitating the development of self-management skills and building self-efficacy is a core feature of chronic pain management.^66^ Self-management is a difficult to measure complex concept,^67^ but typically involves the key skills of problem‐solving, decision making, seeking, and using resources, forming partnerships with their health care providers and taking action.^68^ Acceptance of the persistent nature of pain is a key step in moving from a search for a diagnosis and medical solution to an individualized self-management approach.

A recent randomized controlled trial (n=102) of patients with chronic pain incorporated pain neurophysiology education, cognitive behavioral principles, and individualized, goal-oriented exercises with the type and amount of exercise was based on the participants’ goals, abilities, and pain sensitivity.^69^ Results showed improved function, pain intensity, pain knowledge, catastrophizing, self-efficacy, satisfaction with health care, and global rating of change, but no improvement in pain interference, work status, fatigue, depressive symptoms, or health care utilization in comparison with usual care. By contrast, generic self-management interventions have been shown to have limited effectiveness for patients with chronic MSK pain.^70^

### Patient education

Patient education is a core component of the management of MSK pain. Patient education often reflects that pain is not a true representation of the actual state of the tissues, but it is the nervous system's interpretation of the threat of their injury, which in turn is subject to modulation by various psychological factors, including fear avoidance, catastrophizing, expectations, cognitions, and beliefs. Systematic reviews and meta-analyses on pain science education in chronic MSK pain populations have reported evidence for improving pain ratings, pain knowledge, disability, pain catastrophizing, kinesiophobia, attitudes regarding pain, and physical movement.^71^ However, to achieve clinically important improvements, education should be combined with physical interventions.^72^ Less research has been undertaken on those with acute pain; 1 systematic review reported inconclusive evidence for the benefits of perioperative pain science education on post-operative pain, which can be influenced by health care professionals’ beliefs.^72^

### Managing comorbidities

Patient assessment and management for MSK conditions should always be considered within the context of their general health. By mid-century, 1 in 6 people globally will be aged 65 years or older,^73^ with the prevalence of comorbidities increasing with age. In addition to aging, several other important risk factors are associated with the development of chronic disease, such as lifestyle factors (smoking, alcohol, lack of PA). Many of these factors can cause multiple diseases and many symptoms may have shared underlying neurobiology.^74^ For instance, depression is a common comorbidity in patients with chronic pain and depression itself is associated with a higher incidence of co-morbid somatic illnesses, especially cardiovascular diseases, type 2 diabetes, and metabolic syndrome. In the future, it will be necessary to accommodate, and potentially treat, such comorbidities within pain-related rehabilitation approaches. Systematic reviews and meta-analyses have provided strong evidence for the efficacy of therapeutic exercise for a range of outcomes in patients with a broad range of long-term conditions.^75^

## Implementing patient-centered care

Patient centeredness in MSK care includes effective communication, individualized treatment, working with patient-defined goals, education, and information sharing during all aspects of treatment that facilitates decision making, along with self-management support.^43^

### Behavioral change

A process of behavioral change is the key to successful management of MSK conditions including pain.^37^ Several behavioral change models such as Bandura's self-efficacy theory. Self-efficacy is a person's particular set of beliefs that determine how well one can execute a plan of action in prospective situations.^76^ People's beliefs in their efficacy are developed by 4 main sources of influence: (i) mastery experiences (performance outcomes), (ii) vicarious experiences (social role models), (iii) social persuasion, and (iv) emotional states. If patients and health care professionals contribute to this process and agree on treatment decisions, the process of behavior change is enhanced, and the likelihood of improving pain-related outcomes increases.^37^

In a systematic review on behavior change techniques (BCTs) associated with adherence to prescribed exercise in patients with persistent MSK pain, a moderate level of evidence to support adherence for 5 BCTs was found including (i) social support (unspecified), (ii) goal setting (behavior), (iii) instruction of behavior, (iv) demonstration of behavior, and (v) behavior practice/rehearsal.^77^ For exercise or PA interventions to have a longer-term effect, they need to be enjoyable and meaningful to the individual.^78^^,^^79^

It is also suggested that a pain neuroscience education program may be needed to prime patients for an active lifestyle, remove barriers, and bridge the intention-behavior gap to actively self-manage their problem through a tailored programme.^38^

### Self-management

Successful self‐management including the ability to manage symptoms, treatment, physical, psychological and social consequences, and lifestyle changes related to one's chronic condition is essential in MSK care. There is evidence to support self-management interventions for a variety of different pain conditions^80^ and the use of digital communication-based technology (internet based, telephone supported, virtual reality) may provide innovative options for patients living with chronic pain.^81^ Several mobile-health applications also show promise for (cognitive) treatment^82^ and relapse prevention.^83^

People who are knowledgeable about their condition are better able to self-manage and also deal with others who do not understand their condition;^84^ however, this requires a good level of health literacy. Incorporating effective health literacy strategies into treatment, for example, offering information in bit-size chunks, using plain language and techniques such as the Teach Back methods, have been shown to be effective.^85^

Patients with chronic pain experience exacerbations of their pain problems and relapses may be due to an individual physical event, or it may result from cumulative physical and psychological stresses that challenge patients’ coping resources. Rehabilitation professionals can help to identify situations that are challenging and help patients develop strategies to cope with them. Strategies may include setting criteria to visit health professionals, using pain medication, or briefly resting and relaxing. Plans for resuming activity following an exacerbation are critical.^86^ Technological applications, such as apps, virtual reality, or telephone-based interventions may help patients maintain the skills they learned in their pain management programs and prevent relapse.

## Future directions

### The evolving biopsychosocial model

Although not specific to pain, variations and updates of the BPS model have recently been described in light of new knowledge. The “holistic biopsychosocial model of illness” makes explicit the range of factors that may influence behavior and disability and aims to provide a comprehensive understanding of illness and a rational approach to rehabilitation.^87^ The “Biopsychosocial-Pathways model” describes causal pathways among biological, psychological, and social factors.^88^ Aside from integrating newer interpretations of the BPS model, addressing the many barriers to the adoption of existing methods, such as those linked to the professional knowledge and skills of clinicians (eg, a perceived lack of knowledge of psychosocial factors and/or how to identify or manage them), may promote improved understanding and application of BPS approaches in future clinical practice and education.^89^

### Focusing on the social perspective

Social prescribing initiatives are viewed as a way of addressing the wider social determinants of health and targeting those most socially disadvantaged.^90^ The negative physical and psychological effect of chronic pain is well established, and there is evidence that chronic pain is associated with loneliness and perceived insufficiency of social support.^91^ The use of non-drug, community-based, non-clinical interventions has been proposed as a cost-effective alternative to help those with long-term conditions, including chronic pain, to manage their symptoms and improve their health and well-being.^92^ Social prescription is widely promoted as a way of targeting socioeconomically deprived populations in need of direct health care intervention by linking patients in primary care with support services embedded within the community.^93^ There are a range of social prescription initiatives widely used such as “Arts on Prescription”; “Books on Prescription”; “Education on Prescription”, but “exercise prescription” is 1 of the most used social prescription interventions for the promotion of PA.^94^ While more development in this area is needed, participants in social prescribing programs have reported improvement in outcomes relevant to those with chronic pain such as psychological well-being and positive mood; reduction in anxiety and depression, improvements in physical health, increased self-esteem and confidence, and a reduction in visits to general practitioners.^94^

This article has reviewed current concepts in the management of MSK pain. EBP remains the underlying approach of course, and new evidence is emerging. The BPS model underpins the need to address all aspects of the patient's problem, biomedical issues, psychosocial issues, and the context and environment in which the patient lives and works are all equally important. Reflecting a greater awareness of the BPS approach to MSK issues, psychological informed physical therapy is an emerging area of practice and there is a growing body of evidence to support this practice, particularly within a risk stratification approach to assessment and treatment. Some fundamentals of MSK care remain unchanged, effective communication with the patient as a partner in care is critical. PA and exercise interventions in MSK care remain a core intervention; however, there is a greater awareness of the need to support behavioral change and the techniques required to do this. Likewise, there is a greater awareness of the role of supported self-management and the use of technology as an adjunct to treatment. Public approaches and social prescribing interventions in MSK care are in their infancy, but given the challenges of access to services and treatment no doubt these approaches will grow and develop.

### Musculoskeletal care in 2050

The basic International Classification of Functioning, Disability and Health (ICF) is robust enough to imagine it could still be relevant in 2050 as a framework for defining needs of the individual across the lifespan. Perhaps there will be a more widespread understanding of the influence of environmental factors that could focus attention on new ways of addressing people's needs. For example, attention to the importance of societal attitudes and norms about chronic pain may lead to more public education campaigns like pain revolution (painrevolution.org) and flippin pain (flippinpain.co.uk).

The expanding influence of social media as a source of information in society may become increasingly important. Selective use of social media and information sources can create “echo chambers”, magnify the problem and spreading false/inaccurate information that reinforce attitudes and beliefs, potentially hindering successful pain management. A growing challenge therefore is to ensure that evidence-based messages and information achieve cut-through to the public consciousness.

The ubiquity of digital technology in health care has been accelerated by the COVID-19 pandemic and will influence patient care in the future.^66^ The enforced shift to virtual consultation and treatment, while previously available, was not widely used in clinical practice. However, for many people with chronic pain the logistical benefits of this approach may remain attractive, and the use of virtual appointments may be more desirable. As these solutions lead to great improvements in patient care, the health and wellbeing effects for patients will change the profession. The challenge going forward will be to optimize the important elements of the skilled clinical encounter (communication, empathy, therapeutic touch, and therapeutic alliance). Other technological advances including virtual reality, artificial intelligence, and machine learning will also deepen our understanding of a person's pain and provide personalized treatment approaches.

This article has considered a number of contemporary issues and future directions related to MSK pain. It will be fascinating to observe how changes in pain science and practice develop and affect patient care in the future. Will physical therapists be well-placed to meet the needs of people with persistent pain in 2050? Given the current evidence and future directions of physical therapy practice, perhaps the question would be more usefully rephrased—under what circumstances would physical therapists not be best placed to meet the needs of people with persistent pain in 2050?
